# Multilocus identification and genetic enhancement of *Trichoderma* spp. for entomopathogenic activity against *Spodoptera littoralis*

**DOI:** 10.1186/s12934-025-02834-6

**Published:** 2025-09-12

**Authors:** Nehal A. Atta, Abdelmegid I. Fahmi, Khalid S. Abdel-Lateif, Hesham H. Nagaty, Enas M. Abd EL-Ghany

**Affiliations:** Genetics Department, Faculty of Agriculture, Menoufia University, Shibin El-Kom, Egypt

**Keywords:** *Trichoderma* spp., *Spodoptera littoralis*, Entomopathogenic fungi, Biocontrol, Protoplast fusion, Chitinase activity, Multilocus sequence analysis, Sustainable pest management

## Abstract

**Background:**

The Egyptian cotton leafworm (*Spodoptera littoralis*) is a highly destructive, pesticide-resistant pest affecting over 80 economically important crops across the Mediterranean and African regions. While chemical insecticides offer temporary relief, their long-term use poses environmental and health risks, and resistance development reduces their effectiveness. Biological control using entomopathogenic fungi, particularly *Trichoderma* spp., offers a sustainable alternative. Traditionally, it is used against plant pathogens, *Trichoderma harzianum*, *T. viride*, *T. asperellum*, and *T. longibrachiatum* have also shown insecticidal potential through the production of compounds like peptaibols, gliotoxins, and chitinases, and by inducing systemic resistance in plants. However, the entomopathogenic potential of native *Trichoderma* isolates in Egypt remains undiscovered, and field performance is often inconsistent. This study aims to identify and evaluate native *Trichoderma* strains against *S. littoralis* and enhance their biocontrol efficacy through interspecific protoplast fusion a promising parasexual technique for strain improvement.

**Results:**

Multilocus sequence analysis targeting the *tef1-α* and *rpb2* genes identified the isolates as *T. harzianum*, *T. asperellum*, and *T. longibrachiatum*. Phylogenetic analysis clustered the isolates into three well-distinctive clades corresponding to these species. Among the tested isolates, Tricho19 (*T. longibrachiatum*), Tricho5 (*T. asperellum*), and Tricho30 (*T. harzianum*) demonstrated the highest extracellular chitinase activity and larval mortality in oral bioassays against *S. littoralis*. Interspecific protoplast fusion led to the generation of fusants with significantly enhanced chitinase production and insecticidal activity relative to their parental strains. Greenhouse assays confirmed the superior performance of fusant Fus8, which exhibited the highest larval mortality and antifeedant activity, closely approaching the efficacy of a chemical insecticide.

**Conclusion:**

Interspecific protoplast fusion significantly improved the entomopathogenic performance of *Trichoderma* strains against *S. littoralis*. The enhanced activity of fusant strains, particularly Fus8, highlights the potential of this cost-effective strategy to generate improved biocontrol agents. These findings contribute to the development of sustainable pest management alternatives that can reduce reliance on chemical pesticides in agriculture.

## Background

In attempting to establish sustainable agricultural practices, biological control has emerged as a key research field aimed at reducing dependency on synthetic pesticides. Biological control involves the use of living organisms particularly microorganisms such as entomopathogenic fungi to manage insect pests and plant diseases in an environmentally safe manner. This strategy is gaining global importance due to increasing concerns about pesticide residues, ecological imbalances, and the development of resistant pest populations [1, 2].

Among the most destructive lepidopteran pests affecting a wide range of economically important crops is the Egyptian cotton leafworm, *Spodoptera littoralis* (Boisduval). This polyphagous insect attacks over 80 plant species including cotton, maize, tomato, alfalfa, and legumes, making it a serious threat to agricultural productivity in Egypt and across the Mediterranean and African regions. Its high fecundity, migratory nature, and resistance to multiple classes of insecticides have made it particularly difficult to control [3]. *S. littoralis* is also listed as an A2 quarantine pest by the European and Mediterranean Plant Protection Organization (EPPO, 2023), reflecting its status as a high-risk invasive species.

Conventional chemical pesticides, though effective in the short term, pose long-term threats to human health, beneficial insects, soil quality, and non-target organisms [4]. Furthermore, *S. littoralis* has developed resistance to organophosphates, pyrethroids, and insect growth regulators, highlighting the urgent need for alternative control strategies [5].

In recent years, fungi of the genus *Trichoderma* have gained attention for their dual roles in both plant disease control and insect pest suppression. Traditionally known as biocontrol agents against soilborne phytopathogens, several *Trichoderma* species, including *T. harzianum*, *T. viride*, *T. asperellum*, and *T. longibrachiatum*, have also demonstrated entomopathogenic activity [6, 7].

*Trichoderma* spp. produce insecticidal compounds such as peptaibols, gliotoxins, and chitinases that degrade the insect cuticle and disrupt molting [8, 9]. They can also induce systemic resistance (ISR) in plants, activating defense genes and increasing tolerance to pest attack [10, 11]. Before deployment in pest management, strain efficacy against the target insect must be rigorously assessed under controlled and semi-field conditions. Accurate species-level identification is equally critical, with multilocus analyses incorporating *tef1-α* and *rpb2* sequences now the standard for precise identification [12, 13].

Moreover, native *Trichoderma* isolates adapted to local agroecological conditions tend to exhibit better rhizosphere competence, colonization ability, and endophytic potential compared to exotic strains. These traits contribute to improved plant growth, enhanced nutrient acquisition, and increased tolerance to both biotic and abiotic stresses [14–16].

Despite these advantages, there remains a notable research gap regarding the entomopathogenic potential of native *Trichoderma* isolates from Egypt, particularly against *S. littoralis*. Furthermore, although some strains show promising insecticidal properties, their performance under field conditions is often inconsistent. There is also a lack of research on the genetic enhancement of *Trichoderma* strains to improve their efficacy. One promising strategy is protoplast fusion, a parasexual method that enables the combination of desirable traits from different strains, potentially leading to novel hybrids with superior biocontrol performance [17].

This study addresses these gaps by isolating and identifying native *Trichoderma* strains, evaluating their insecticidal activity against *S. littoralis*, and applying interspecific protoplast fusion to enhance their biocontrol efficacy under both laboratory and greenhouse conditions.

## Materials and methods

### Molecular identification of Trichoderma isolates

DNA was extracted from 31 isolates previously isolated from our laboratory [16] following the protocol of [18]. To enable accurate species identification of *Trichoderma*, partial fragments of the translation elongation factor 1-α (Tef1) and RNA polymerase II large subunit (RPB2) genes were amplified. PCR was performed in a total volume of 25 µL, containing 12.5 µL of PCR Master Mix (Promega Corp., Madison, WI, USA), 1 µL each of forward and reverse primers (100 µM) for Tef1 and RPB2, 1 µL of genomic DNA template (100 ng/µL), and nuclease-free water to complete the final volume. PCR cycling conditions are detailed in Table 1.

**Table 1:** PCR primers and amplification program

Target	Primers	Initial Denaturation	35 cycles		
			Denaturation	Annealing (15 s)	Extension
Tef1	EF1 (ATGGGTAAGGARGACAAGAC)	95 °C 5 min	95 °C 15 s	55 °C	72 °C 1 min
	EF2 (GGARGTACCAGTSATCATGTT)				
RPB2	fRPB2-5f (GAYGAYMGWGATCAYTTYGG)			54 °C	
	fRPB2-7cr (CCCATRGCTTGTYYRCCCAT)				

#### Sequence analysis

PCR products were purified using the QIAquick^®^ PCR Purification Kit (QIAGEN, Hilden, Germany) according to the manufacturer’s instructions. Bidirectional Sanger sequencing was performed by Beijing Liuhe BGI Gene Technology Co., Ltd. (Beijing, China). The raw sequence data were quality-checked, trimmed, and assembled into consensus sequences using Sequencher^®^ v5.4.6 (Gene Codes Corp., Ann Arbor, MI, USA). Assembled sequences were submitted to GenBank (NCBI) to obtain accession numbers, and species identification was verified using BLASTn analysis against the NCBI nucleotide database.

The Tef1 and RPB2 sequences were aligned using MUSCLE [19] and manually edited using BioEdit v7.2.5 [20]. Phylogenetic analyses were conducted using the Maximum Likelihood (ML) method in MEGA X [21], with branch support assessed via 1000 bootstrap replicates. Resulting trees were visualized and annotated using FigTree v1.4.4.

### Chitinase activity assay

#### Quantitative chitinase activity

Colloidal chitin was prepared according to [22]. The detection medium per liter contained: 0.3 g MgSO₄·7 H₂O (magnesium sulfate heptahydrate), 3.0 g (NH₄)₂SO₄ (ammonium sulfate), 0.3 g KH₂PO₄ (potassium dihydrogen phosphate), 1.0 gm citric acid monohydrate, 15 g agar, 200 µL Tween-80, 4.5 g colloidal chitin, and 0.15 g bromocresol purple (BCP). Finally, the pH was adjusted to 4.7 and the medium sterilized by autoclaving at 121 °C for 15 min. *Trichoderma* culture plugs were inoculated into the medium and incubated at 28 °C. Purple zones formation indicated chitinase activity [23].

#### Quantitative chitinase activity

Quantitative analysis followed the method of Agrawal and Kotasthane [23]. Culture plugs were inoculated into the same broth medium (without BCP) containing colloidal chitin and incubated at 28 °C, 200 rpm for 5 days. The culture was filtered (Whatman No. 1), and filtrates were stored at -20 °C. To quantify reducing sugars released from colloidal chitin, a reaction mixture (1 mL culture supernatant, 0.3 mL sodium acetate buffer (1 M, pH 4.6) and 0.2 mL colloidal chitin) was incubated at 40 °C for 20 h, then centrifuged at 13,000 rpm at 6 °C for 5 min. Supernatants (0.75 mL) were mixed with 0.25 mL DNS reagent and 0.1 mL NaOH (10 M), then heated at 100 °C for 5 min. After cooling, absorbance was measured at 575 nm. A standard curve using N-acetyl-β-D-glucosamine (NAGA) was used to calculate chitinase activity expressed in international units (IU).

$$ \begin{gathered} {\text{Chitinase activity}}\left( {{\text{IU}}} \right)\left( {\mu {\text{mol}}/{\text{mL}}/{\text{min}}} \right){\text{ }} \hfill \\ = \frac{{\begin{array}{*{20}c} {{\text{Amount}}~{\text{of}}~{\text{NAGA}}} \\ {{\text{released}}~\left( {\mu {\text{mol}}} \right)} \\ \end{array} }}{{\begin{array}{*{20}c} {{\text{Volume}}~{\text{of}}~{\text{enzyme}}} \\ {{\text{used}}~\left( {{\text{mL}}} \right)} \\ \end{array} }} \times \begin{array}{*{20}c} {{\text{Incubation}}} \\ {{\text{time}}~\left( {{\text{min}}} \right)} \\ \end{array} ~ \hfill \\ \end{gathered} $$

The amount of N-acetyl-β-D-glucosaminidase (NAGA) released was quantified using a standard curve derived from absorbance measurements at 575 nm. In this essay, 1 mL of culture supernatant was used as the enzyme source, and the reaction mixture was incubated for 20 h (equivalent to 1200 min) to ensure adequate enzymatic activity.

### Bioassay against cotton leafworm

Third instar larvae of *Spodoptera littoralis* were obtained from the Plant Protection Research Institute (Dokki, Giza, Egypt) and reared on castor oil plant (*Ricinus communis*) leaves under controlled environmental conditions (25 ± 1 °C, 50 ± 5% relative humidity, and a 16:8 h light: dark photoperiod). For bioassays, larvae were placed in plastic containers (25 × 16 × 10 cm) covered with fine mesh to ensure adequate ventilation and prevent escape.

For spore suspension preparation, *Trichoderma* spores were collected from 7-day-old PDA cultures using 5 mL sterile water with 0.1% Tween 80 per plate. The suspension was filtered through sterilized cheesecloth to remove debris. Spore concentrations were determined using a hemocytometer and adjusted to 10², 10⁶, and 10⁸ spores/mL, then stored at 4 °C. However, for filtrate preparation, *Trichoderma* cultures were grown in 50 mL of potato dextrose broth (PDB) on a rotary shaker at 150 rpm for 7 days at 28 °C. The resulting cultures were filtered through sterilized cheesecloth, and the filtrates were subsequently diluted with sterile distilled water at ratios of 1:1 and 1:2 (v/v).

Finally, topical and oral bioassays were conducted according to the method of [24], with slight modifications. These modifications included adjustment of spore concentration e.g. 1 × 10⁷ spores/mL. Use of both spore suspensions and culture filtrates as separate treatments. Addition of 0.1% Tween 80 to improve spore dispersion. Inclusion of moist filter paper to maintain optimal humidity inside the containers. Daily replacement of treated leaves in the oral assay to ensure consistent exposure. For the topical application, third instar larvae were either sprayed with or dipped into the spore suspension or culture filtrate for 30 s. Treated larvae were then air-dried and transferred to sterilized plastic containers. For the oral application, castor oil plant (*Ricinus communis*) leaves were immersed in the respective treatments, air-dried, and provided as food to the larvae. Control treatments consisted of sterile distilled water supplemented with 0.1% Tween 80. All larvae were incubated in darkness at 25 ± 2 °C for 10 days, and mortality was recorded daily.

### Protoplast fusion and fusant selection

Antifungal agents (fluconazole, terbinafine, itraconazole, and nystatin) were tested at various concentrations on potato dextrose agar (PDA) supplemented with 0.1% Triton X-100 to determine minimum inhibitory concentrations (MICs). Based on these results, terbinafine and nystatin were selected as selectable markers to distinguish isolates MNF-NAH-Tricho5 and MNF-NAH-Tricho30, respectively.

Spore suspensions (10⁶ spores/mL) of each isolate were inoculated into potato dextrose broth (PDB) supplemented with 1.5% yeast extract and incubated at 28 °C for 16 h on a rotary shaker at 160 rpm. The resulting spores were filtered through sterile cheesecloth and washed sequentially with sterile distilled water, phosphate buffer (pH 5.7), and an osmotic stabilizer solution (0.6 M KCl).

Mycelia were then digested using lysing enzymes at a concentration of 20 mg/mL and incubated at 28 °C for 3 h at 100 rpm to release protoplasts. The protoplast suspension was filtered, washed with STC buffer (1.2 M sorbitol, 10 mM Tris-HCl, and 50 mM CaCl₂; pH 7.5), and adjusted to a final concentration of 10⁶ protoplasts/mL.

Protoplast viability was assessed by plating 10³ protoplasts on regeneration medium (PDA supplemented with 0.6 M KCl, 0.01% Triton X-100, and the appropriate antifungal marker). Viability was calculated using the following formula [25]:

$${\text{Viability }}\left( \% \right){\text{ }}={\text{ }}\left( {{\text{Nc }}/{\text{ Np}}} \right) \times {\text{1}}00$$

where N_p_ is the number of inoculated protoplasts and N_C_ is the number of resulting colonies.

Equal volumes of protoplast suspensions from the two selected isolates were fused using 40% polyethylene glycol (PEG) 6000 in STC buffer, incubated at room temperature for 10 min, and centrifuged. The resulting fused protoplasts were plated on PDA containing both antifungal markers to select for successful fusants. Control (non-fused) protoplasts were plated in parallel. Putative fusants were sub-cultured for five successive generations on colloidal chitin agar supplemented with the respective antifungal agents to confirm mitotic stability, following the method of [26].

### Greenhouse evaluation of Trichoderma strains and fusants against Spodoptera littoralis

Greenhouse experiments were conducted to evaluate the efficacy of *Trichoderma* strains and their fusants against third instar larvae of *Spodoptera littoralis* on cabbage (*Brassica oleracea* var. capitata). Cabbage seedlings were grown in sterilized soil composed of 50% sand, 30% clay, and 20% peat moss, in 35 cm plastic pots. After 30 days of growth, 10 larvae were introduced to each plant.

*Trichoderma* treatments were applied as spore suspensions at a concentration of 1 × 10⁸ spores/mL. Control plants were sprayed with sterile distilled water containing 0.1% Tween 80. A chemical control was included using Lambda-Cyhalothrin 5% as a foliar spray. Each pot was covered with a plastic mesh cage to prevent larval escape and allow ventilation. Environmental conditions were maintained at 25 ± 5 °C.

The experiment followed a randomized complete block design with three replicates per treatment. Treatment details are presented in Table 2.

**Table 2:** *Trichoderma* (as an entomopathogen) applied against 3rd instar larvae of cotton leafworms under greenhouse conditions

No.	Treatment	No.	Treatment
1	Plant only (Negative control)	10	Plant + MNF-NAH-Tricho5 + Larvae
2	Plant + Larvae	11	Plant + MNF-NAH-Tricho30 + Larvae
3	Plant + MNF-NAH-Tricho5	12	Plant + MNF-NAH-Tricho19 + Larvae
4	Plant + MNF-NAH-Tricho30	13	Plant + Fusant (3) + Larvae
5	Plant + MNF-NAH-Tricho19	14	Plant + Fusant (7) + Larvae
6	Plant + Fusant (3)	15	Plant + Fusant (8) + Larvae
7	Plant + Fusant (7)	16	Plant + C.P + Larvae
8	Plant + Fusant (8)		
9	Plant + Chemical pesticide (C.P)		

Observations and data collection were carried out over a period of 10 days.

$${\text{Larval Mortality }}\left( \% \right){\text{ }}={\text{ }}\left( {{\text{D }}/{\text{ I}}} \right) \times {\text{1}}00$$

Where *I* = initial larval number; *D* = number of dead larvae.

$$ {\text{Antifeedant Index }}\left( {{\text{AFI}}} \right){\text{ }} = {\text{ }}\left[ {\frac{{\left( {{\text{C }} - {\text{ T}}} \right)}}{{\left( {{\text{C }} + {\text{ T}}} \right)}}} \right]{\text{ }} \times {\text{ 1}}00 $$

Where *C* = leaf area consumed in the control, *T* = leaf area consumed in the treatment.

### Data analysis

Data were analyzed using one-way analysis of variance (ANOVA) in CoStat software v6.311 (Cohort Software, Monterey, CA, USA). Treatment means were compared using Duncan’s multiple range test at a significance level of *P* < 0.05.

Phylogenetic analyses were performed using the Maximum Likelihood method implemented in MEGA version 11, based on [27]. Bootstrap consensus trees were generated from 1000 replicates, with positions containing gaps or missing data excluded from the analysis.

## Results

### Molecular identification of Trichoderma spp.

#### PCR amplification, sequencing, and sequence analysis

Partial sequences of the translation elongation factor 1-alpha (*tef1*) gene, specifically including the fourth intron, and the second largest subunit of RNA polymerase II (*rpb2*) were successfully amplified using PCR and subsequently sequenced to enable accurate species identification of the *Trichoderma* isolates. Sequence analysis revealed that the isolates belonged to three species: *Trichoderma harzianum*, *T. asperellum*, and *T. longibrachiatum*. All obtained gene sequences were submitted to the NCBI GenBank database, and the corresponding accession numbers are listed in Table 3. Among the identified isolates, *T. harzianum* was the most predominant (23 isolates), followed by *T. asperellum* (6 isolates), and *T. longibrachiatum* (2 isolates).

**Table 3:** Molecular identification of *Trichoderma* isolates based on *Tef1* and *RPB2* gene sequences

Isolate Code	GenBank Accession Number (Tef1)	GenBank Accession Number (RPB2)	Species name
MNF-NAH-Tricho1	PP556982	PP566714	*Trichoderma harzianum*
MNF-NAH-Tricho2	PP556983	PP566715	*Trichoderma harzianum*
MNF-NAH-Tricho3	PP556984	PP566716	*Trichoderma harzianum*
MNF-NAH-Tricho4	PP556985	PP566717	*Trichoderma harzianum*
MNF-NAH-Tricho5	PP556986	PP566718	*Trichoderma asperellum*
MNF-NAH-Tricho6	PP556987	PP566719	*Trichoderma harzianum*
MNF-NAH-Tricho7	PP556988	PP566720	*Trichoderma harzianum*
MNF-NAH-Tricho8	PP556989	PP566721	*Trichoderma asperellum*
MNF-NAH-Tricho9	PP556990	PP566722	*Trichoderma asperellum*
MNF-NAH-Tricho10	PP556991	PP566723	*Trichoderma harzianum*
MNF-NAH-Tricho11	PP566693	PP566724	*Trichoderma harzianum*
MNF-NAH-Tricho12	PP566694	PP566725	*Trichoderma harzianum*
MNF-NAH-Tricho13	PP566695	PP566726	*Trichoderma asperellum*
MNF-NAH-Tricho14	PP566696	PP566727	*Trichoderma harzianum*
MNF-NAH-Tricho15	PP566697	PP566728	*Trichoderma harzianum*
MNF-NAH-Tricho 16	PP566698	PP566729	*Trichoderma harzianum*
MNF-NAH-Tricho17	PP566699	PP566730	*Trichoderma harzianum*
MNF-NAH-Tricho18	PP566700	PP566731	*Trichoderma asperellum*
MNF-NAH-Tricho19	PP566701	PP566732	*Trichoderma longibrachiatum*
MNF-NAH-Tricho20	PP566702	PP566733	*Trichoderma harzianum*
MNF-NAH-Tricho21	PP566703	PP566734	*Trichoderma harzianum*
MNF-NAH-Tricho22	PP566704	PP566735	*Trichoderma harzianum*
MNF-NAH-Tricho23	PP566705	PP566736	*Trichoderma harzianum*
MNF-NAH-Tricho24	PP566706	PP566737	*Trichoderma asperellum*
MNF-NAH-Tricho25	PP566707	PP566738	*Trichoderma harzianum*
MNF-NAH-Tricho26	PP566708	PP566739	*Trichoderma harzianum*
MNF-NAH-Tricho27	PP566709	PP566740	*Trichoderma harzianum*
MNF-NAH-Tricho28	PP566710	PP566741	*Trichoderma longibrachiatum*
MNF-NAH-Tricho29	PP566711	PP566742	*Trichoderma harzianum*
MNF-NAH-Tricho30	PP566712	PP566743	*Trichoderma harzianum*
MNF-NAH-Tricho31	PP566713	PP566744	*Trichoderma harzianum*

#### Phylogenetic analysis

Sequences of both *tef1* and *rpb2* genes were aligned with reference sequences obtained from the NCBI database and used to construct phylogenetic trees. For the *tef1* dataset, reference sequences of *T. harzianum* (OP026354), *T. longibrachiatum* (OP948263), and *T. asperellum* (MN329691) were used. For the *rpb2* dataset, reference sequences included *T. harzianum* (OQ026376), *T. longibrachiatum* (OP410935), and *T. asperellum* (PP068268).

**Fig. 1:**
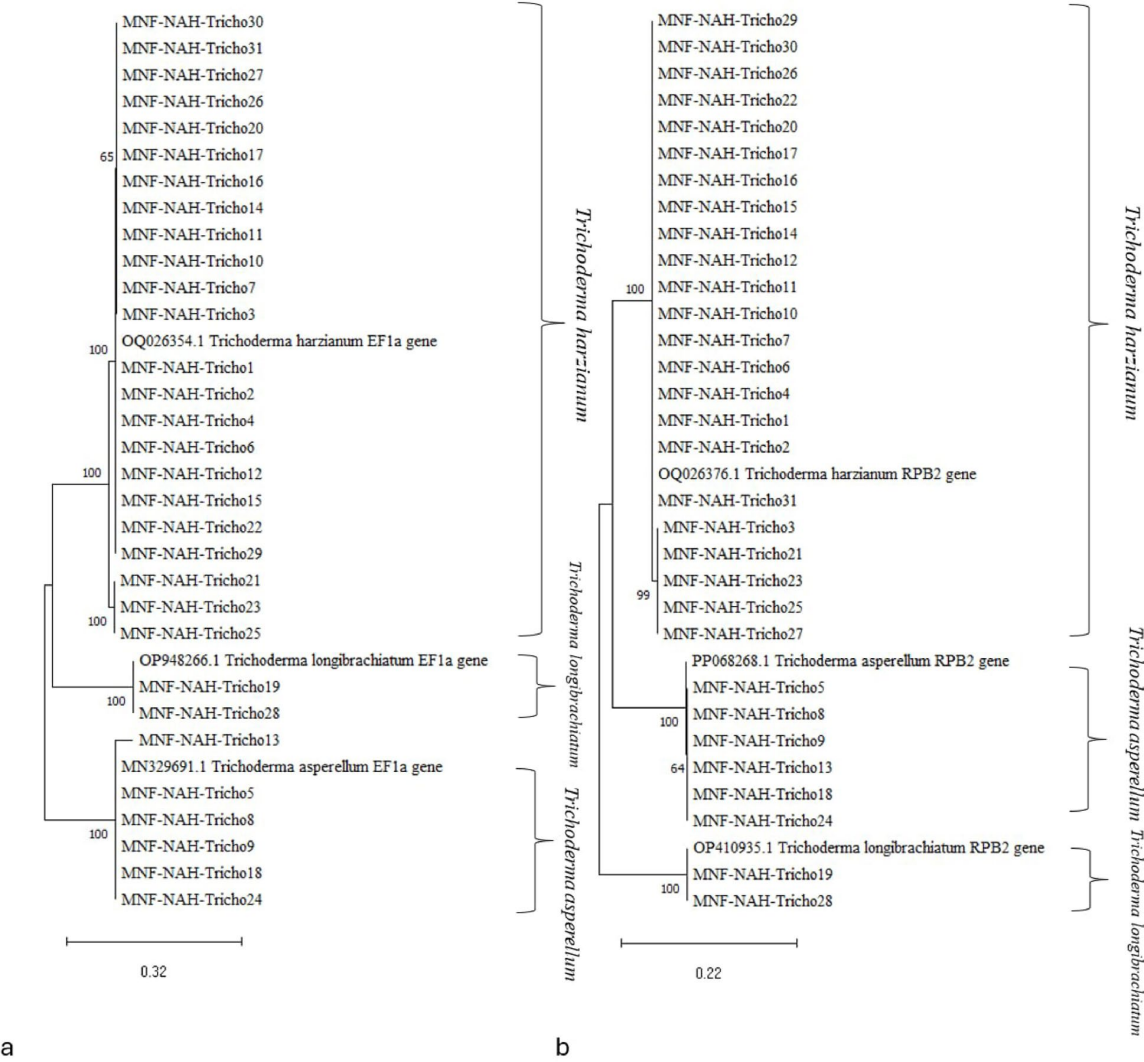
Phylogenetic trees showing the genetic diversity among 31 *Trichoderma* isolates based on DNA sequences of (a) translation elongation factor 1-alpha (tef1-α) and (b) RNA polymerase II second largest subunit (rpb2). Reference strains in each phylogram are denoted by their GenBank accession numbers. Bootstrap support values1,000 replicates) are presented next to the corresponding nodes

Figure 1 illustrates the phylogenetic relationships among the isolates. Bootstrap values were shown at each node to indicate statistical support. The initial trees for heuristic searches were constructed automatically using the Neighbor-Joining and BioNJ algorithms, based on pairwise distances estimated via the [27]. The final topology was selected based on the highest log-likelihood score.

The phylogenetic trees for both *tef1* and *rpb2* genes revealed that the isolates clustered into three well-supported, distinct clades, each corresponding to one of the three identified *Trichoderma* species.

### Biocontrol evaluation of Trichoderma spp

#### Qualitative chitinase activity

**Fig. 2:**
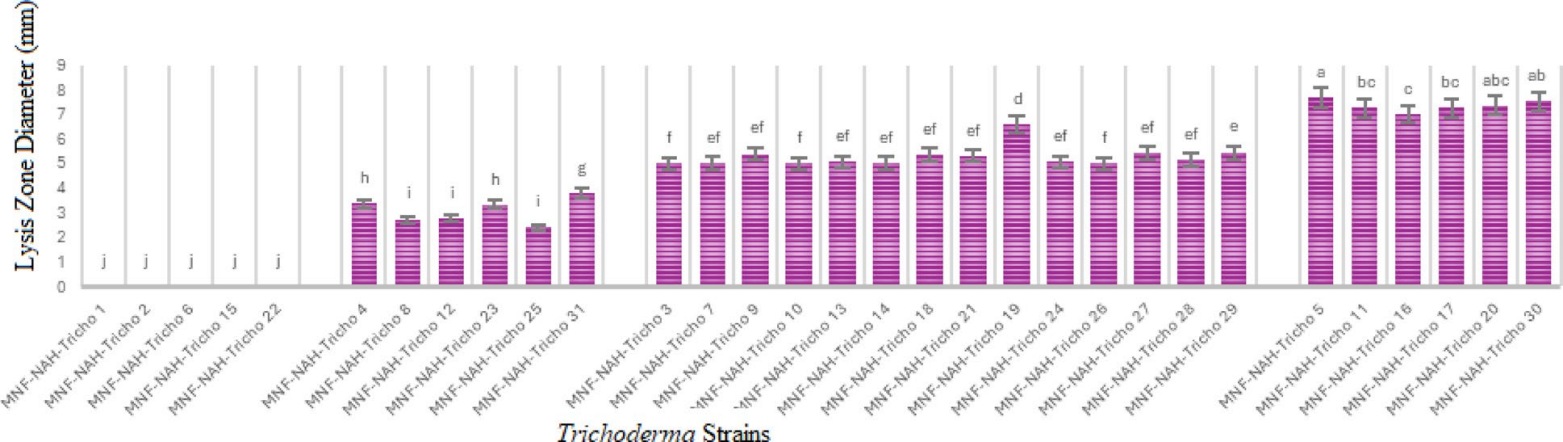
Qualitative chitinase activity: diameter of lysis purple-colored zone of *Trichoderma strains* on solid media. Superscript values indicate Duncan’s grouping means with the same letter are not significantly different. The data are presented as the mean ± standard error

The chitinolytic potential of the *Trichoderma* isolates was evaluated using a detection medium containing bromocresol purple (BCP) dye at pH 4.7. After three days of incubation on colloidal chitin agar, the development of purple zones around the fungal colonies indicated enzymatic degradation of chitin and a subsequent localized increase in pH due to the release of N-acetylglucosamine. The size of these purple zones was measured as an indicator of qualitative chitinase activity (Fig. 2).

Isolates exhibited varying levels of chitinolytic activity; high activity; MNF-NAH-Tricho5, -11, -16, -17, -20, and − 30; moderate activity; MNF-NAH-Tricho3, -7, -9, -10, -13, -14, -18, -19, -21, -24, -26, -27, -28, and − 29; Low activity; MNF-NAH-Tricho4, -8, -12, -23, -25, and − 31 and no detectable activity; MNF-NAH-Tricho1, -2, -6, -15, and − 22. These results indicate significant variation in chitinase production among the tested isolates.

#### Quantitative chitinase activity

To further assess chitinase production, quantitative assays were conducted by measuring the release of reducing sugars from colloidal chitin substrates. The concentration of N-acetylglucosamine (NAGA) released was determined using a standard calibration curve. Chitinase activity was expressed in terms of the amount of NAGA produced per milliliter.

**Fig. 3:**
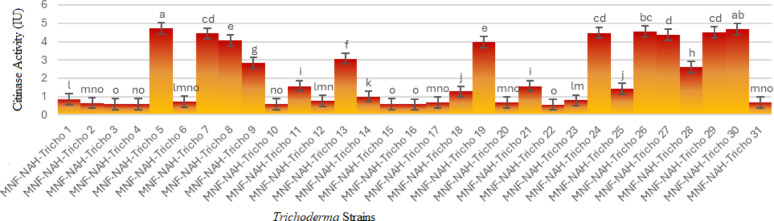
Quantitative chitinase activity: Superscript letters indicate Duncan’s grouping means with the same letter are not significantly different. The data are presented as the mean ± standard error

All *Trichoderma* isolates exhibited measurable chitinolytic activity, although the levels varied substantially. The most active strains included MNF-NAH-Tricho5, MNF-NAH-Tricho30, MNF-NAH-Tricho7, MNF-NAH-Tricho24, MNF-NAH-Tricho26, MNF-NAH-Tricho27 and MNF-NAH-Tricho29. In contrast, MNF-NAH-Tricho16 and MNF-NAH-Tricho22 exhibited the lowest enzymatic activity (Fig. 3). The remaining isolates demonstrated intermediate activity levels.

#### In vitro entomopathogenicity assay

Three highly active *Trichoderma* isolates MNF-NAH-Tricho5 (*T. asperellum*), MNF-NAH-Tricho30 (*T. harzianum*), and MNF-NAH-Tricho19 (*T. longibrachiatum*) were selected to evaluate their entomopathogenic potential against the cotton leafworm (*Spodoptera littoralis*). Two modes of application were tested: topical application and oral ingestion, using both spore suspensions and culture filtrates.

**Fig. 4:**
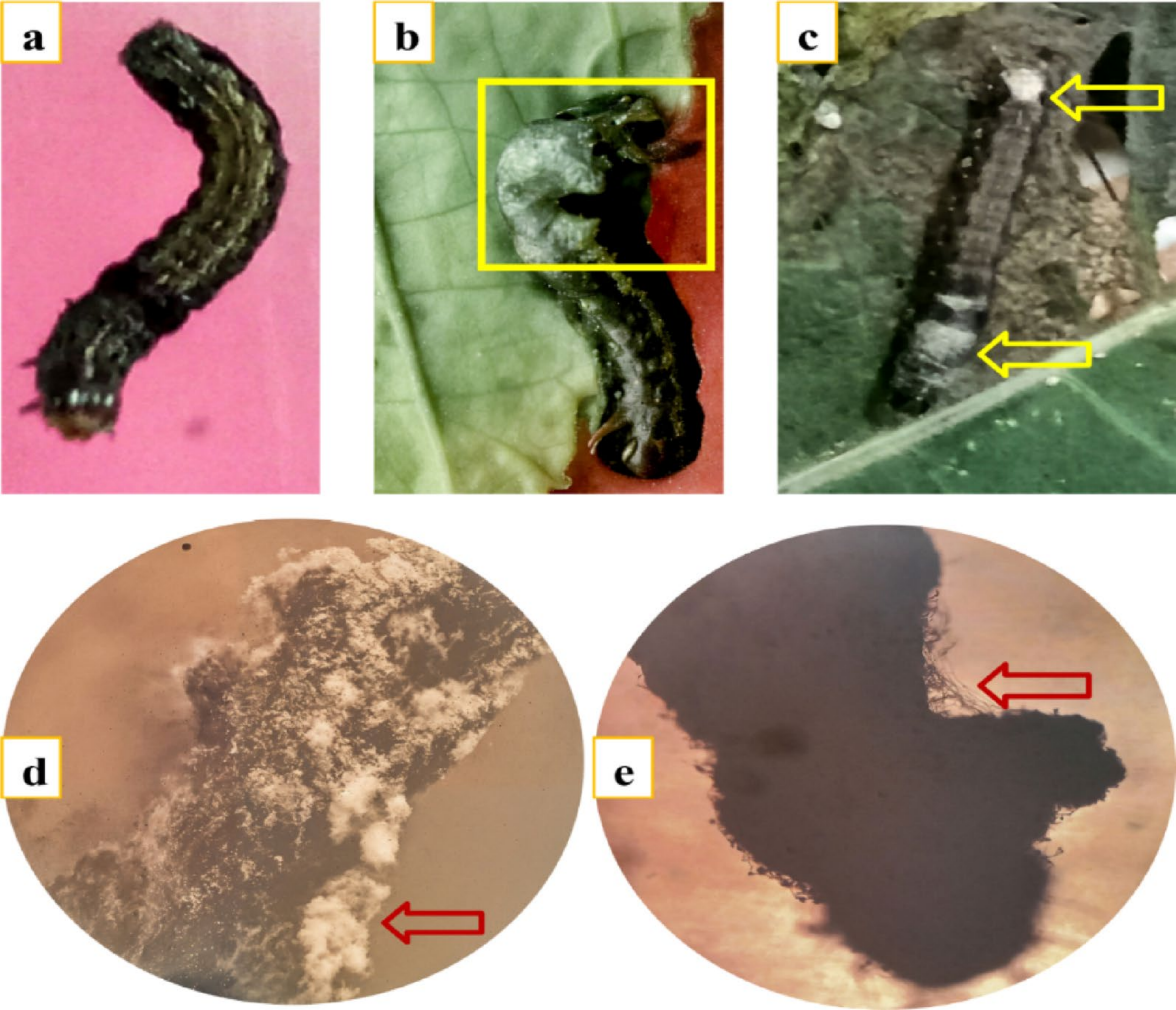
Parasitic effect of *Trichoderma* spp. on *Spodoptera littoralis* larvae. (a) Healthy, untreated larva; (b) colonization of larval body by *Trichoderma asperellum* (MNF-NAH-Tricho5), highlighted within the yellow box; (c) growth of *Trichoderma harzianum* (MNF-NAH-Tricho30) on the larval surface, indicated by yellow arrows; (d, e) stereomicroscope images showing extensive overgrowth of *Trichoderma* spp. on the larval body

Larval mortality was monitored daily for 10 consecutive days. Visual symptoms of infection were observed under a stereomicroscope. Figure 4b and c illustrate parasitic interactions caused by Tricho5 and Tricho30 on days 5 and 6 post-treatment, respectively, while Fig. 4d and e describe hyphal colonization on larval bodies.

**Fig. 5:**
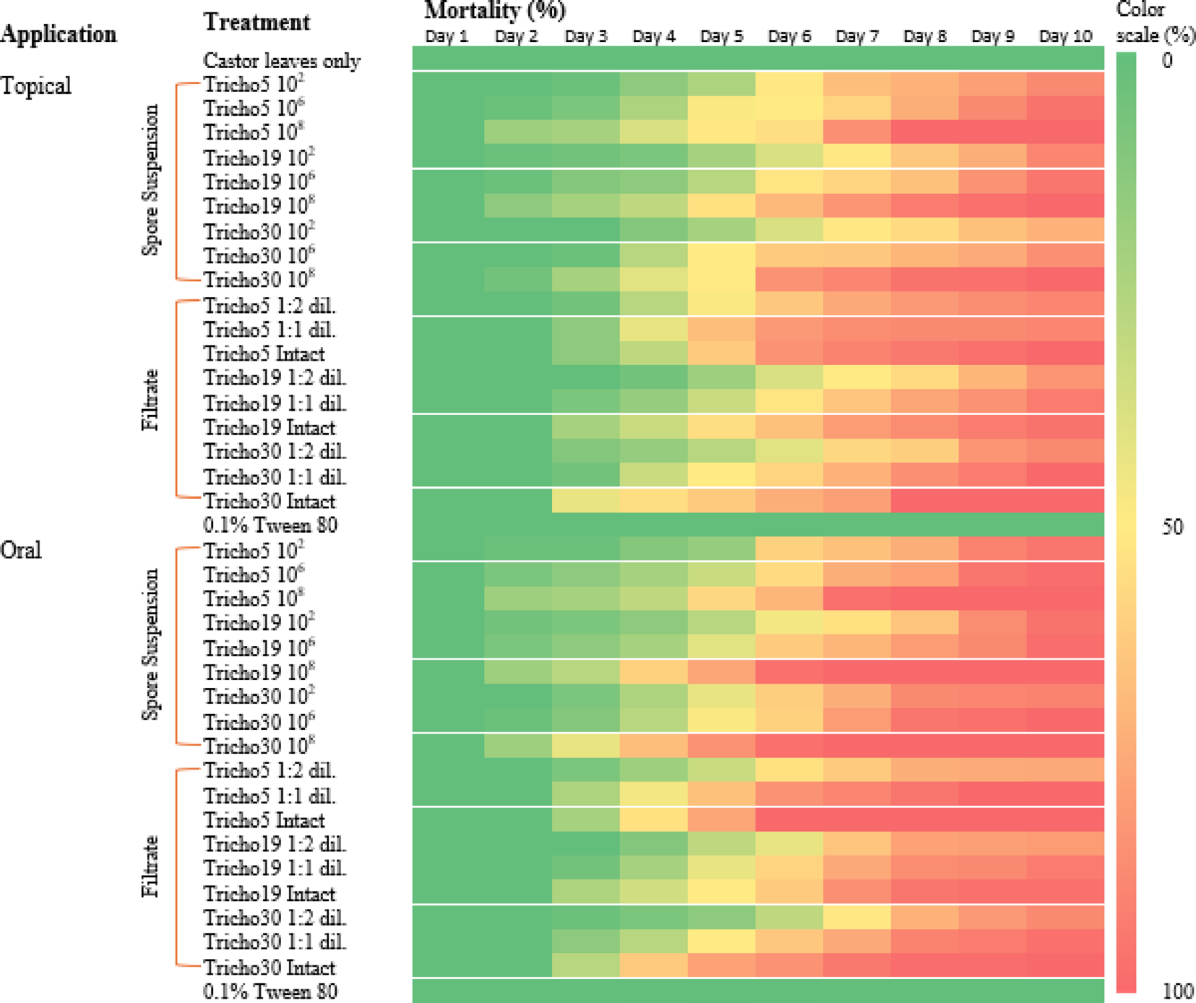
Heatmap illustrates the mortality percentage of cotton leafworm (*S. littoralis)* larvae induced by different *Trichoderma* spp. over ten consecutive days of post-treatment. Two application methods were compared. Color intensity corresponds to mortality rate, with higher intensity indicating increased larval mortality

**Fig. 6:**
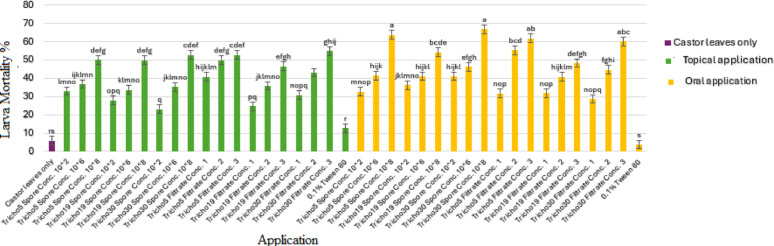
Average mortality percentage of cotton leafworm larvae (*Spodoptera littoralis*) induced by *Trichoderma* spp. using two different application methods over ten days post-treatment. Values represent the mean values of three replicates ± standard error (SE). According to Duncan’s multiple range test (*P* < 0.05), different superscript letters indicate statistically significant differences

A heatmap in Fig. 5 presents a comparative analysis of application methods, revealing that higher treatment concentrations generally resulted in increased mortality. Among treatments, the oral application of spore suspensions was the most effective. Figure 6 summarizes the 10-day average mortality rates, confirming that oral treatments outperformed topical applications, and spore suspensions were more effective than culture filtrates.

### Genetic improvement via interspecific protoplast fusion

To enhance biocontrol efficacy, two highly chitinolytic strains; MNF-NAH-Tricho5 (*T. asperellum*) and MNF-NAH-Tricho30 (*T. harzianum*) were selected for genetic improvement through interspecific protoplast fusion. The fusion process was conducted using an antifungal resistance-based selection strategy to distinguish parental strains from successful fusants.

#### Selection of antifungal markers

Each parent strain was tested for sensitivity to four antifungal agents Itraconazole, Fluconazole, Terbinafine, and Nystatin using concentration range. The tested concentration ranges were as follows: Nystatin (10–250 µg/mL), Terbinafine (8–250 µg/mL), Fluconazole (1–150 µg/mL), and Itraconazole (1–150 µg/mL).The minimum inhibitory concentrations (MICs) were determined as the lowest concentrations that completely inhibited fungal growth. MNF-NAH-Tricho5 was found to tolerate Nystatin up to 150 µg/ml, with complete growth inhibition at 200 µg/ml. Conversely, MNF-NAH-Tricho30 was susceptible to Terbinafine at concentrations ≥ 100 µg/ml. Based on these results, Terbinafine (150 µg/ml) and Nystatin (200 µg/ml) were selected as selective markers for MNF-NAH-Tricho5 and MNF-NAH-Tricho30, respectively.

#### Protoplast isolation and fusion

Protoplasts were generated from actively growing mycelia using a lytic enzyme mixture. Cell wall digestion initiated within one hour of incubation, and complete protoplast release occurred by three hours. The number of viable protoplasts was estimated at; 1.58 × 10⁶ protoplasts/ml for MNF-NAH-Tricho5; 1.79 × 10⁶ protoplasts/ml for MNF-NAH-Tricho30. Protoplast regeneration was conducted on PDA supplemented with Terbinafine and Nystatin, respectively. Viability assessments showed; MNF-NAH-Tricho5 79.8% viability on Terbinafine and MNF-NAH-Tricho30 82.5% viability on Nystatin (Table 4). The fusion of protoplasts was carried out using polyethylene glycol (PEG 6000) via a soft rolling method. The resulting fusants were initially selected on a medium containing 2% colloidal chitin and both antifungal markers. A total of 22 fusants were recovered.

**Table 4:** Number and viability of released protoplasts of *Trichoderma* strains grown on PDA medium supplemented with antifungal agents

Strain	Antifungal used	No. of released protoplast (count/ml)	Viable regenerated protoplasts (%)
MNF-NAH-Tricho5	Terbinafine (150 µg/ml)	1.58 × 10^6^	79.8
MNF-NAH-Tricho30	Nystatin (200 µg/ml)	1.79 × 10^6^	82.5

#### Fusant stabilization and designation

**Fig. 7:**
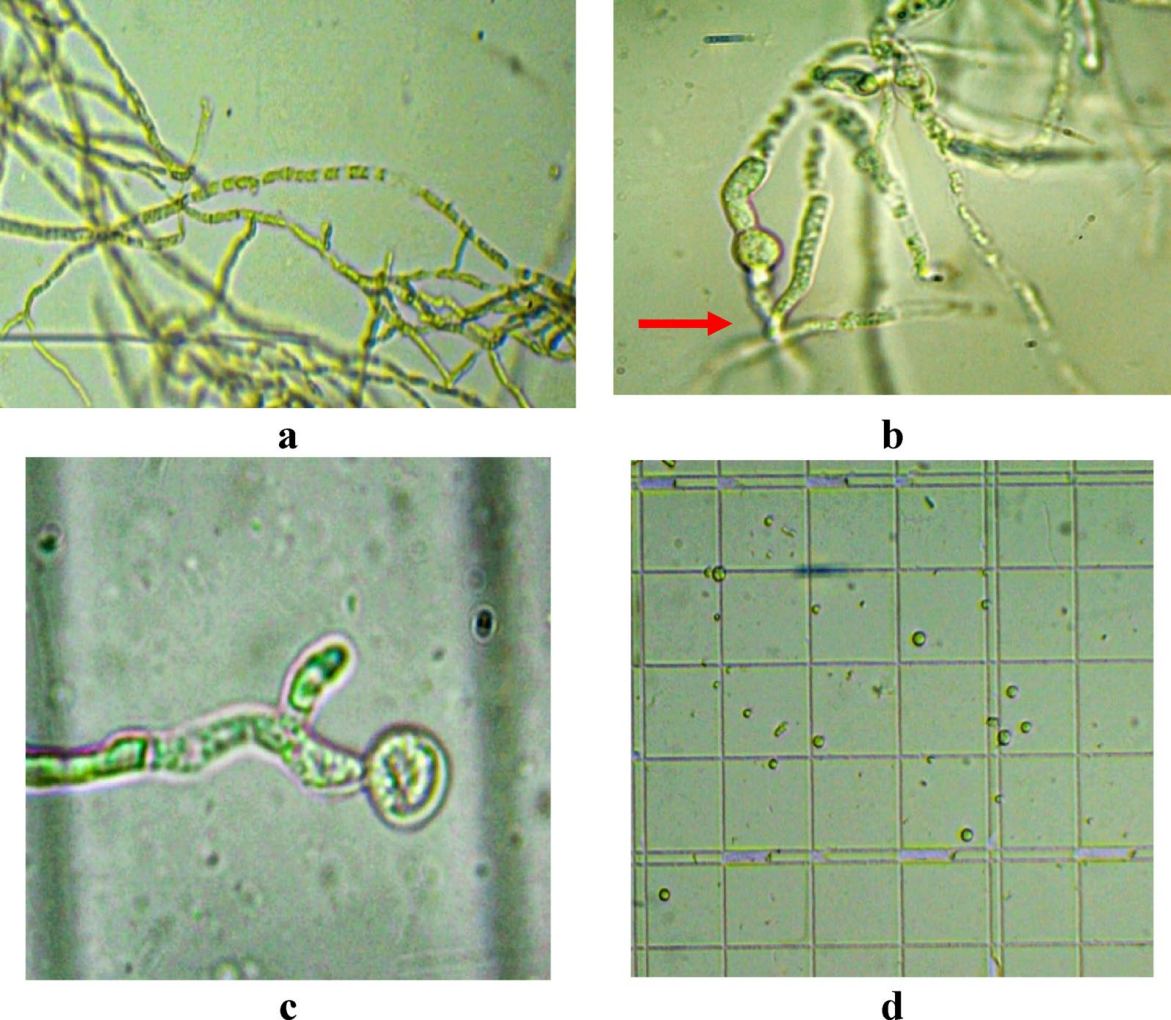
Protoplast isolation steps: **a** Lysis of cell wall, **b** Cell protoplast, **c** Protoplast release, **d** Free protoplasts

**Fig. 8:**
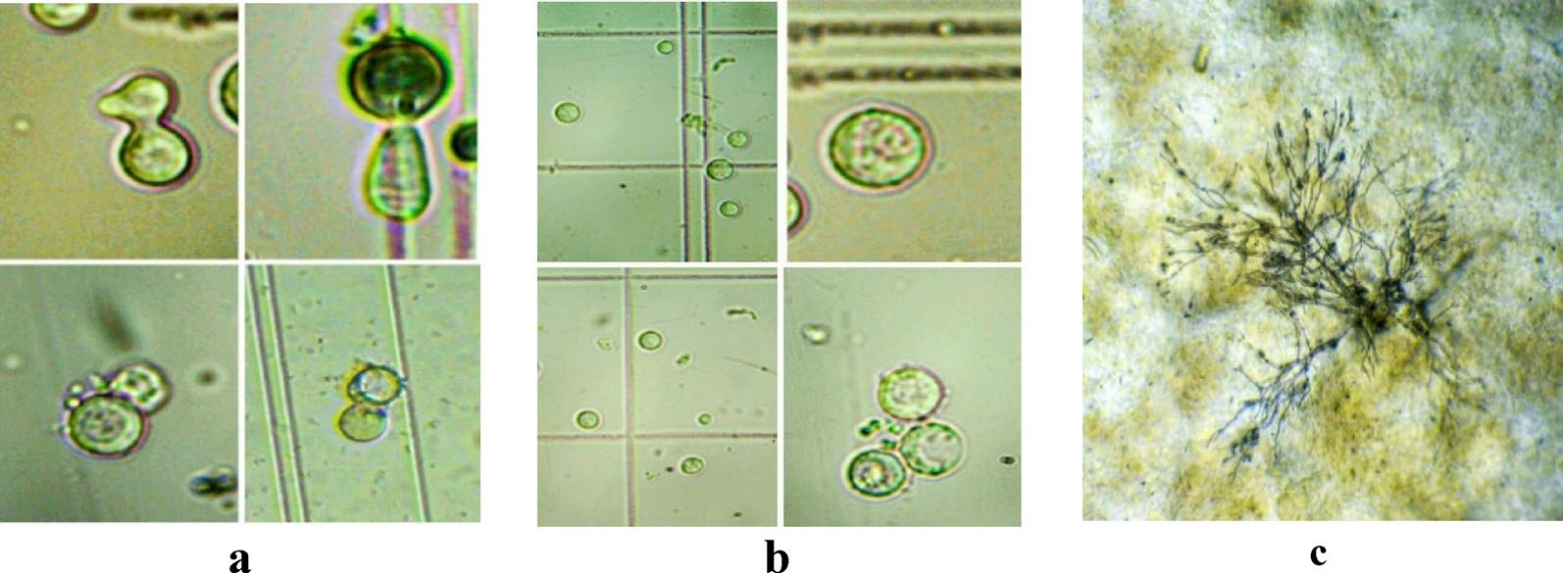
Protoplast fusion process. **a** fusion of protoplast, **b** fused protoplasts, and **c** germination of fused protoplast after 48 h of incubation on selection medium

The fusants were sub-cultured for five successive generations on selective medium to assess genetic stability. Twenty stable fusants were obtained and designated as Fus1 through Fus20. Figure 7 demonstrates the major steps in protoplast isolation and fusion, while Fig. 8 presents the performance and resulting fusants.

### Evaluation of fusants

#### Chitinase activity in parental and fused Trichoderma strains

Both qualitative and quantitative analyses were performed to evaluate the chitinolytic potential of the 20 genetically stable fusants in comparison with their parental strains (*T. asperellum* MNF-NAH-Tricho5 and *T. harzianum* MNF-NAH-Tricho30). Table 5 summarizes the qualitative and quantitative results.

**Table 5:** Qualitative (lysis zone diameter) and quantitative assays (Units/ml) of total chitinase activity for fusants and their parental strains MNF-NAH-Tricho5 (P5) and MNF-NAH-Tricho30 (P30)

Isolate	Qualitative assay*			Quantitative assay		
	Diameter (cm)	% improvement from parents		Unit/ml	% improvement from parents	
P5	5.1 ^gh^			4.651 ^i^		
P30	5.53 ^fg^			4.567 ^j^		
		P5	P30		P5	P30
Fus1	6.53 ^de^	128.04	118.08	4.861 ^de^	104.54	106.37
Fus2	5.07 ^gh^	99.41	91.68	4.788 ^fgh^	102.97	104.77
Fus3	8.77 ^a^	171.96	158.59	5.139 ^a^	110.52	112.45
Fus4	6.4 ^de^	125.49	115.73	4.898 ^cd^	105.33	107.18
Fus5	7.9 ^bc^	154.90	142.86	4.941 ^bc^	106.26	108.12
Fus6	7.6 ^c^	149.02	137.43	4.826 ^ef^	103.78	105.60
Fus7	9 ^a^	176.47	162.75	4.988 ^b^	107.27	109.15
Fus8	8.97 ^a^	175.88	162.21	5.099 ^a^	109.66	111.58
Fus9	4.6 ^h^	90.20	83.18	0.898 ^k^	19.31	19.65
Fus10	5.53 ^fg^	108.43	100.00	4.801 ^fgh^	103.25	105.05
Fus11	6.57 ^d^	128.82	118.81	4.912 ^cd^	105.63	107.48
Fus12	6.7 ^d^	131.37	121.16	4.818 ^efg^	103.61	105.43
Fus13	5.9 ^def^	115.69	106.69	4.783 ^fgh^	102.86	104.66
Fus14	5.73 ^efg^	112.35	103.62	4.906 ^cd^	105.51	107.35
Fus15	5.5 ^fg^	107.84	99.46	4.792 ^fgh^	103.05	104.86
Fus16	6.13 ^def^	120.20	110.85	4.753 ^h^	102.22	104.00
Fus17	8.37 ^ab^	164.12	151.36	4.827 ^ef^	103.81	105.62
Fus18	6.3 ^def^	123.53	113.92	4.929 ^c^	106.00	107.86
Fus19	6.67 ^d^	130.78	120.61	4.767 ^gh^	102.52	104.31
Fus20	6.13 ^def^	120.20	110.85	4.777 ^fgh^	102.73	104.53

Values are the means of three replicates. Different letters in the same column indicate significant differences, according to Duncan’s test (*P* < 0.05)

In the qualitative assay, all fusants except Fus2 and Fus9 exhibited increased chitinase activity relative to the parents. Among them, Fus7 showed the most extensive colloidal chitin degradation after three days of incubation, with improvement rates of 176.5% and 162.8% compared to MNF-NAH-Tricho5 (P5) and MNF-NAH-Tricho30 (P30), respectively. Fus8 and Fus3 also displayed substantial enhancements, with improvement rates of; Fus8 175.9% over P5 and 162.2% over P30 and Fus3 172% over P5 and 158.6% over P30. Conversely, Fus10 demonstrated chitinolytic activity similar to that of the parental strains.

Quantitative assays further supported these findings. Chitinase activity was expressed in units/ml based on N-acetylglucosamine (NAGA) release; Fus3 exhibited the highest activity at 5.14 units/ml, corresponding to an increase of 110.52% over P5 and 112.45% over P30. Fus8 followed closely, with activity improvements of 109.7% over P5 and 111.58% over P30. Fus7 showed enhancements of 107.27% and 109.15% compared to P5 and P30, respectively. Fus9 showed significantly reduced activity, indicating possible loss of function. Overall, fusants exhibited greater enhancement percentages in the qualitative assay, suggesting differences in sensitivity or detection thresholds between the two methods.

#### Entomopathogenic potential of parental and fused strains

##### In vitro evaluation

To evaluate insecticidal potential, the top three chitinase-producing fusants (Fus3, Fus7, and Fus8) were tested against *Spodoptera littoralis* larvae in an oral bioassay. Each fusant, along with its respective parental strain and the previously tested MNF-NAH-Tricho19 (control), was applied to castor leaves and offered to larvae.

**Fig. 9:**
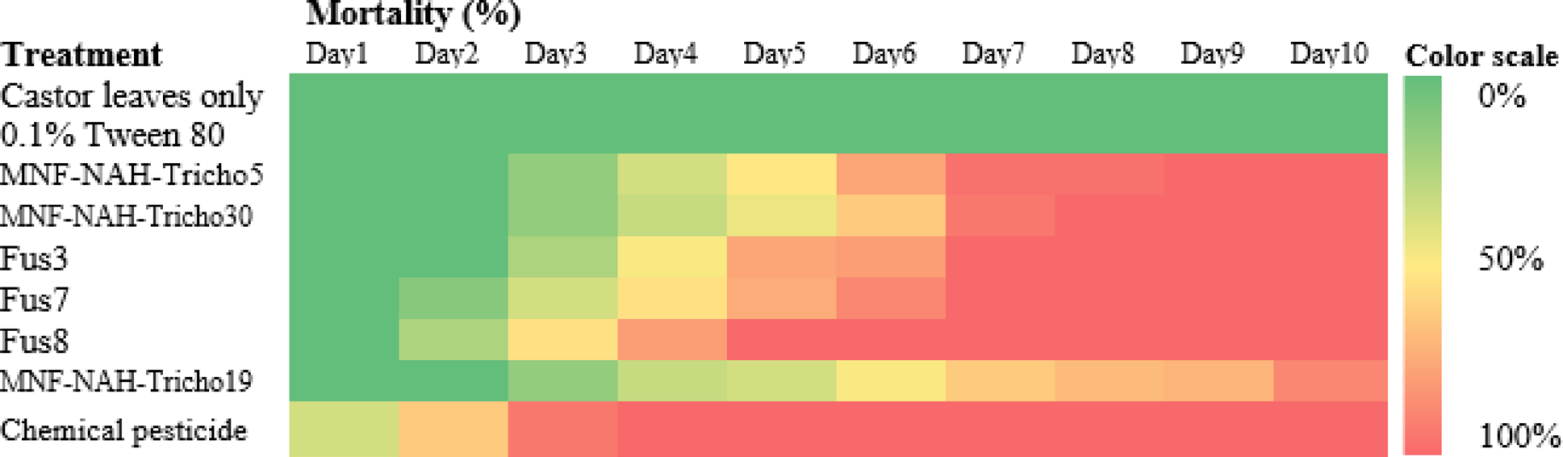
Heatmap of cotton leafworm larval mortality percentage induced by three selected fusants and their parental strains comparing between them by oral application method for ten consecutive days post treatment under laboratory conditions. MNF-NAH-Tricho19 and chemical pesticide were used as control. The color intensity representing application effect of *Trichoderma* strains on *s. littoralis* mortality, as the higher intensity color indicated the higher mortality percentage

**Fig. 10:**
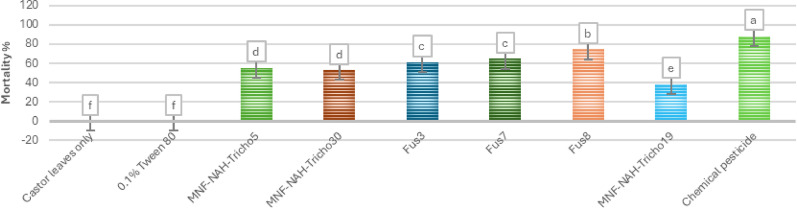
Average mortality percentages of cotton leafworm larva induced by three selected fusants and their parents of ten days after treatment. MNF-NAH-Tricho19 and chemical pesticide were used as control. Values are the means of three replicates. According to Duncan’s test (*P* < 0.05), different superscript letters indicate significant differences. The data are presented as the mean ± standard error

The fusants exhibited a faster onset of larval suppression compared to their parental strains. As shown in the heat map of Fig. 9, growth inhibition became evident by the third day post-treatment, with Fus8 showing early activity within 48 h, followed by Fus7. Peak larval mortality occurred between days 6–10 for most treatments, except for Fus8, which reached peak mortality on day 5. The chemical pesticide used as a positive control induced larval death as early as 24 h post-treatment. Figure 10 illustrates the mean mortality rates over the 10-day period; Fus8 achieved the highest mortality among the *Trichoderma* strains (approx. 74%), second only to chemical control. Fus3 and Fus7 recorded average mortalities of 61% and 64%, respectively. All fusants outperformed their parental strains in larval control efficacy.

##### In vivo evaluation (Greenhouse Trial)

A greenhouse experiment was conducted to assess the efficacy of the selected fusants on cabbage plants infested with *S. littoralis*. The larvae were monitored for ten days following treatment.

**Fig. 11:**
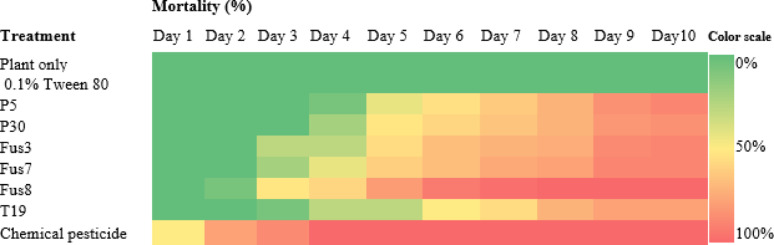
Heatmap of cotton leafworm larval mortality percentage induced by three selected fusants and their parental strains comparing between them by oral application method for ten consecutive days post treatment on cabbage plants under greenhouse conditions. MNF-NAH-Tricho19 and chemical pesticide were used as control. The color intensity representing application effect of *Trichoderma* strains on *s. littoralis* mortality, as the higher intensity color indicated the higher mortality percentage

**Fig. 12:**
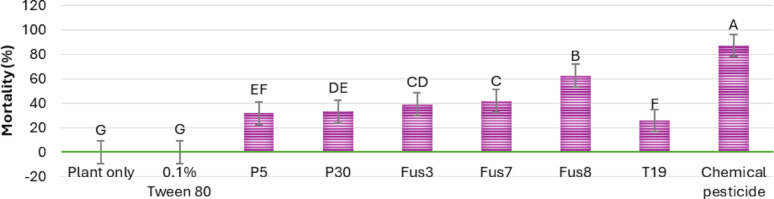
Average mortality percentages of cotton leafworm larva induced by three selected fusants and their parents of ten days after treatment. MNF-NAH-Tricho19 and chemical pesticide were used as control. Values are the means of three replicates. According to Duncan’s test (*P* < 0.05), different superscript letters indicate significant differences. The data are presented as the mean ± standard error

As expected, the chemical pesticide produced the most rapid and pronounced larval suppression. However, the fusants also showed significantly improved performance relative to their parental strains; Fus8 triggered a noticeable decline in larval population within 72 h. Fus7 and Fus3 showed significant reductions by the fourth and fifth days, respectively. Figure 11 heatmap presents a heatmap of larval mortality over time, while Fig. 12 summarizes the average mortality across treatments; Fus8 again achieved the highest mortality among the biological treatments (about 62%), followed by Fus7 (42%) and Fus3 (41%). In contrast, the parental strains MNF-NAH-Tricho5 and MNF-NAH-Tricho30 induced only 31% and 33% mortality, respectively.

**Fig. 13:**
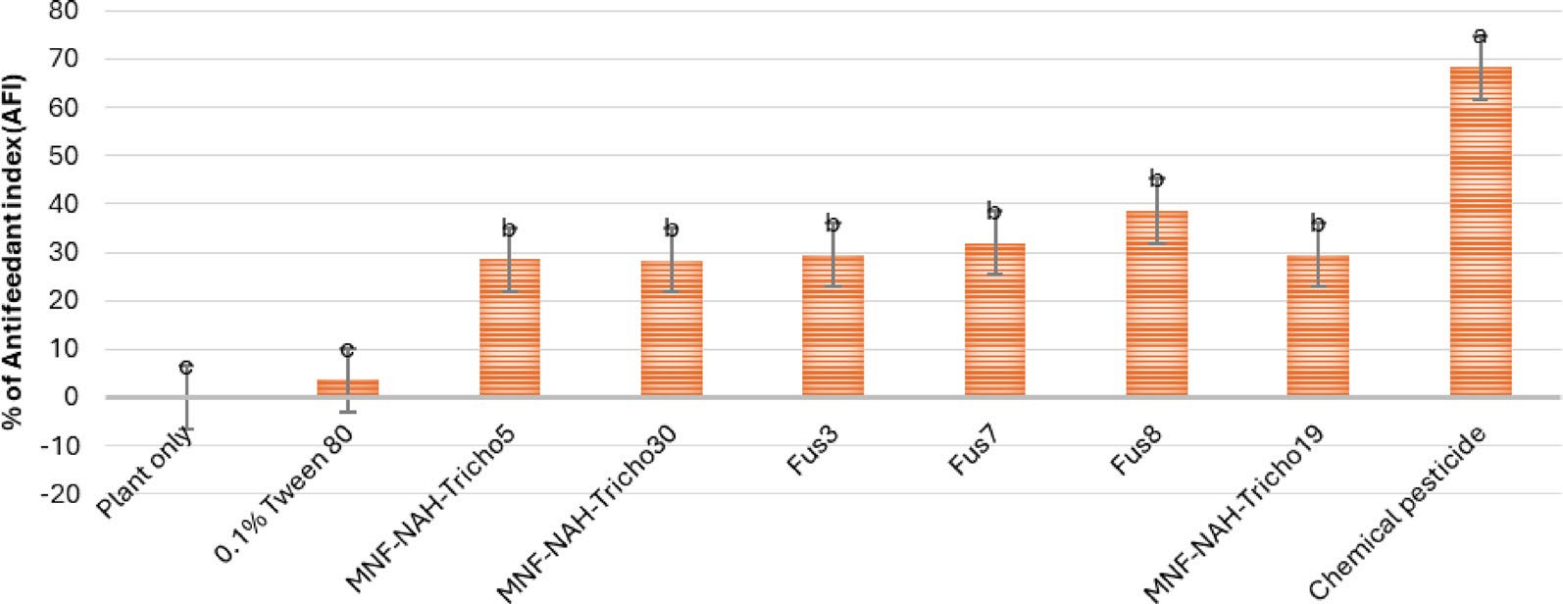
Antifeedant indexes of *S. littoralis* third-instar larvae exposed to various treatments of *Trichoderma* spore suspensions on cabbage ten days after treatment. MNF-NAH-Tricho19 and chemical pesticide were used as control. Values are the means of three replicates. According to Duncan’s test (*P* < 0.05), different superscript letters indicate significant differences. The data are presented as the mean ± standard error

**Fig. 14:**
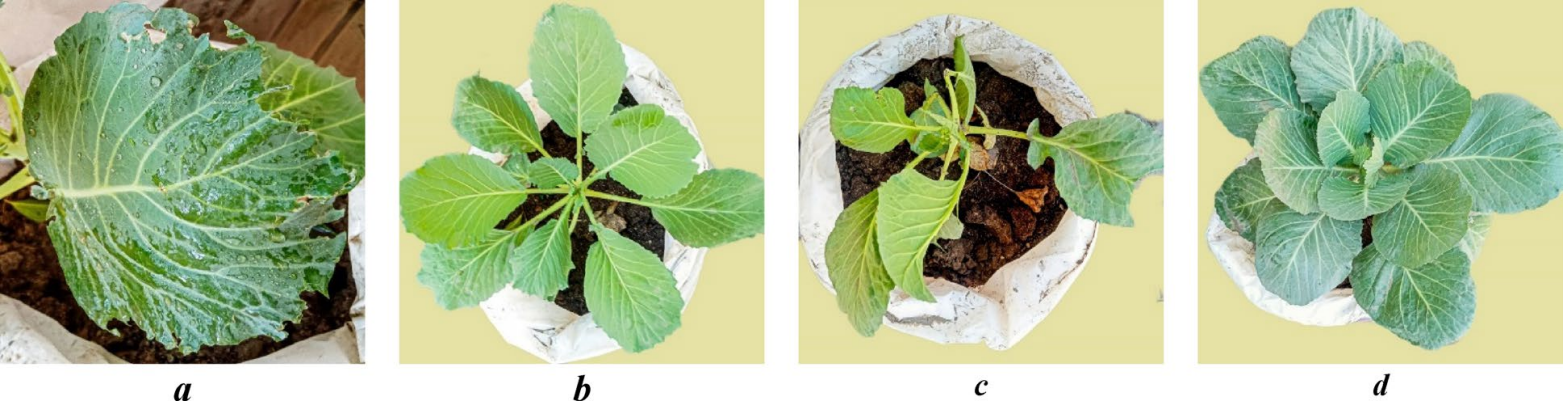
Cabbage treated with *Trichoderma* by oral application, **a** disease symptoms of cotton leafworm on cabbage leaves, **b** untreated cabbage plant, **c** infected cabbage plant with *S. littoralis*, and **d** treated cabbage plant with Fus8 and infected

To assess indirect effects, an antifeedant assay was conducted. As shown in Fig. 13, Fus8-treated plants exhibited the highest antifeedant index (39%), indicating strong feeding deterrence. Other fusants and parent strains ranged from 28 to 32%. Figure 14 documents larval feeding damage, typically starting from lower leaves and progressing upward. Fus8-treated plants exhibited visibly enhanced vigor and reduced foliar damage (Fig. 14d), contrasting with the untreated control (Fig. 14a). This suggests a potential plant growth-promoting effect in addition to pest suppression.

## Discussion

### Molecular identification and phylogenetic resolution

Species identification in *Trichoderma* is often depended on insufficient morphological characterization [28, 29]. While ITS regions are widely used as a primary barcode, they often lack resolution at the species level. In this study, multilocus sequence analysis employing *tef1-α* and *rpb2* loci enabled accurate differentiation among isolates, identifying them as *T. harzianum*, *T. asperellum*, and *T. longibrachiatum*. Phylogenetic reconstruction using series of sequences generated three distinct, well-supported clades corresponding to these species.

The dominance of *T. harzianum* and *T. asperellum* aligns with their reported adaptability, stress flexibility, and rhizospheric competitiveness [30, 31]. Low intraspecific genetic variation among isolates suggests a conserved population structure, which may limit natural variability in traits relevant to biocontrol. This highlights the importance of applying strain enhancement techniques to expand their functional capabilities.

### Chitinase activity and screening for entomopathogenic potential

Chitinases are key virulence factors in fungal entomopathogens, mediating hydrolysis of chitin in the insect cuticle and peritrophic membrane [32]. All tested isolates exhibited chitinase activity, though Tricho5 (*T. asperellum*) and Tricho30 (*T. harzianum*) showed the highest levels in both qualitative and quantitative assays.

These results confirmed chitinase activity as a useful functional biomarker for selecting strains with insecticidal potential. Furthermore, the findings support the hypothesis that hydrolytic enzyme production contributes significantly to fungal virulence against insect pests.

### Bioassay against Spodoptera littoralis larvae

The Egyptian cotton leafworm (*S. littoralis*) poses a severe challenge to agricultural productivity due to its polyphagy and resistance to conventional pesticides [33]. Bioassays with top-performing isolates (Tricho5, Tricho19, and Tricho30) demonstrated dose-dependent and method-dependent mortality. Oral application of spore suspensions consistently outperformed topical treatments, likely due to direct interaction with the insect gut.

Among treatments, Tricho30 spore suspension showed the fastest onset of mortality, achieving > 85% larval death within six days. Filtrate treatments were also effective, suggesting that secondary metabolites contributed to larvicidal activity. Control treatments produced minimal mortality (< 10%), confirming the specificity of fungal action. These findings are consistent with previous reports on oral delivery effectiveness [24].

### Protoplast fusion for strain enhancement

To enhance biocontrol potential, protoplast fusion was performed between Tricho5 and Tricho30. High protoplast viability (> 79%) facilitated successful fusion, resulting in 20 genetically stable fusants selected on dual-antibiotic media. This classical genetic tool enabled the recombination of desirable traits, namely, high chitinase production and virulence beyond what is achievable through conventional selection.

Fusants showed distinct phenotypes and improved chitinolytic profiles, supporting previous studies on the effectiveness of protoplast fusion in enhancing fungal performance [34–36]. This technique remains a powerful platform for strain improvement when transformation systems or CRISPR tools are unavailable.

### Fusant performance: laboratory and greenhouse assessment

Among the fusants, Fus8 exhibited the most potent biocontrol effect. In laboratory assays, it achieved 100% larval mortality by day 5, significantly exceeding the parental strains and closely approximating chemical pesticide performance (complete mortality by day 4). In greenhouse trials, Fus8 induced progressive mortality from day 8 to day 10. Though slower than chemical treatments, its average mortality rate was only about 20% lower.

Importantly, Fus8 showed a high antifeedant index (61%), indicating both toxic and deterrent effects. This dual mechanism may involve fungal metabolites interacting with insect chemoreceptors [37–41]. Such activity supports its use not only as a bioinsecticide but also as a crop-protective agent.

## Conclusion

Despite the recognized potential of entomopathogenic fungi, their large-scale adoption remains constrained by inconsistent field performance and slower pest suppression relative to synthetic pesticides [42]. This study highlights the potential of protoplast fusion as an effective strategy to enhance the entomopathogenic activity of *Trichoderma* spp. Notably, the fusant strain *Fus8* exhibited superior biocontrol efficacy, outperforming parental isolates and closely matching chemical treatments in larval mortality and feeding deterrence. These findings demonstrate that strain improvement via protoplast fusion can significantly bridge the efficacy gap between biological and chemical control agents. Future research should focus on assessing the stability, formulation compatibility, and broad-spectrum activity of these improved strains under diverse agro-ecological conditions to enable their integration into sustainable pest management systems.

## Data Availability

No datasets were generated or analysed during the current study.

## Abbreviations

ITS: Internal transcribed spacer
Tef 1α: Translation elongation factor 1-alpha gene
Rpb2: DNA-directed RNA polymerase II core large subunit
PDA: Potato Dextrose Agar
BCP: Bromocresol Purple
NAGA: N-acetyl-β-D-glucosamine
